# Letter to the editor: Time to consider unusual or severe headache and fatigue as indicator symptoms for COVID-19 testing?

**DOI:** 10.2807/1560-7917.ES.2022.27.1.2101188

**Published:** 2022-01-06

**Authors:** Alma Tostmann

**Affiliations:** 1 Department of Medical Microbiology, Radboud Centre for Infectious Diseases, Nijmegen, the Netherlands

**Keywords:** diagnostic testing, COVID-19, testing strategy, public health, infection control

**To the editor:** With great interest I read the article by Brandal et al. about an outbreak caused by the severe acute respiratory syndrome coronavirus 2 (SARS-CoV-2) Omicron variant following a company Christmas party in Oslo, Norway on 26 November 2021 [1]. The authors shared very relevant information regarding the early symptoms of the 66 confirmed and 15 probable cases associated with this outbreak. Among the 81 cases, the most common symptoms were cough (83%), followed by runny/stuffy nose (78%), fatigue/lethargy (74%), sore throat (72%), headache (68%) and fever (54%).

In many European countries, the symptoms fatigue/lethargy and headache are not an indication for SARS-CoV-2 testing. It is thus important to know the profile of early symptoms of those infected with the Omicron and future variants. Firstly, how many cases had reported only fatigue, lethargy and/or headache as initial symptoms? These people may not have come forward for coronavirus disease (COVID-19) testing under regular circumstances (non-outbreak setting). And secondly, how many cases with fatigue, lethargy and/or headache as initial symptoms eventually developed symptoms that are indicative of COVID-19 after a few days? In the first example, no case isolation and quarantine of contacts would have occurred. In the second example, case-isolation would have occurred albeit rather late, and their close contacts with a subsequent infection would have probably already started their infectious period.

Having a detailed and thorough early symptom profile among vaccinated and unvaccinated young and healthy individuals is crucial, given the high infectiousness of the Omicron variant, due to immune escape [2]. With this information, current testing guidelines could be reviewed in order to ensure that targeted preventive measures such as isolation and quarantine remain effective in prevention of ongoing transmission.

Which symptoms would be the best indicators for COVID-19 testing may differ by country. Data from the ‘Covid Symptoms Study’ showed that the top five symptoms reported in the app for Omicron infection were runny nose, headache, fatigue, sneezing, and a sore throat [3]. Influenza-like symptoms such as severe headache, muscle aches, fatigue and lethargy have been associated with a positive SARS-COV-2 test in the first pandemic wave already, including among healthcare workers in March to April 2020 [4], as well as among adults and children during SARS-CoV-2 wild-type and Alpha variant dominance [5], and among school-aged children in the United Kingdom [6]. However, these symptoms are not included in most countries’ COVID-19 test guidelines.

When including a symptom as an indicator for testing, the prevalence in the population should also be considered, not only the positive predictive value and association with the test outcome. Headache and fatigue are very common symptoms. When not described specifically, they may be too sensitive and increase testing demand unnecessarily without adding much to case-finding. So when considering these symptoms as indication for testing, they should be defined very specifically, for example with adjectives such as ‘uncommon, unusual, sudden and/or severe’.

I would therefore like to call on scientists and public health professionals who report on early COVID-19 symptoms to specify the proportion of cases who reported non-respiratory symptoms such as fatigue, lethargy and/or headache as their only symptoms. This could be helpful in supporting potential improvements in screening or diagnostic strategies. On the other hand, awareness of the availability of resources remains important as well. In addition, we all need to remain vigilant for novel symptoms with the emergence of new SARS-CoV-2 variants.

## References

[1] Brandal LT MacDonald E Veneti L Ravlo T Lange H Naseer U Outbreak caused by the SARS-CoV-2 Omicron variant in Norway, November to December 2021. Euro Surveill. 2021;26(50). 10.2807/1560-7917.ES.2021.26.50.2101147 34915975 PMC8728491

[2] Ferguson N, Ghani A, Hogan A, Hinsley W, Volz E. Report 49 - Growth, population distribution and immune escape of Omicron in England. London: MRC Centre for Global Infectious Disease Analysis; 2021. Available from: https://www.imperial.ac.uk/mrc-global-infectious-disease-analysis/covid-19/report-49-Omicron/

[3] Iacobucci G . Covid-19: Runny nose, headache, and fatigue are commonest symptoms of omicron, early data show. BMJ. 2021;375(3103):n3103. 10.1136/bmj.n3103 34916215

[4] Tostmann A Bradley J Bousema T Yiek WK Holwerda M Bleeker-Rovers C Strong associations and moderate predictive value of early symptoms for SARS-CoV-2 test positivity among healthcare workers, the Netherlands, March 2020. Euro Surveill. 2020;25(16):2000508. 10.2807/1560-7917.ES.2020.25.16.2000508 32347200 PMC7189649

[5] Elliott J Whitaker M Bodinier B Eales O Riley S Ward H Predictive symptoms for COVID-19 in the community: REACT-1 study of over 1 million people. PLoS Med. 2021;18(9):e1003777. 10.1371/journal.pmed.1003777 34582457 PMC8478234

[6] Molteni E Sudre CH Canas LS Bhopal SS Hughes RC Antonelli M Illness duration and symptom profile in symptomatic UK school-aged children tested for SARS-CoV-2. Lancet Child Adolesc Health. 2021;5(10):708-18. 10.1016/S2352-4642(21)00198-X 34358472 PMC8443448

